# Prevention with Sublay Mesh of Trocar Site Hernia (PRESUME): Study Protocol for a Multicenter Randomized Controlled Trial

**DOI:** 10.3389/jaws.2026.16067

**Published:** 2026-03-27

**Authors:** Emma Sánchez-Sáez, Enric Sebastian-Valverde, Ramon Vilallonga Puy, Ana Ciscar Bellés

**Affiliations:** 1 Parc Sanitari Sant Joan de Déu, España, Spain; 2 Universitat Autonoma de Barcelona, Barcelona, Spain; 3 Hospital Universitari Vall d'Hebron, Barcelona, Spain; 4 Hospital de la Santa Creu i Sant Pau, Barcelona, Spain

**Keywords:** hernia prevention, laparoscopic cholecystectomy, prophylactic mesh, sublay repair, trocar site hernia

## Abstract

**Background:**

Trocar Site Incisional Hernia (TSIH) is the most common postoperative complication after laparoscopic surgery, with incidences exceeding 30% in high-risk patients. Despite advances in minimally invasive approaches, optimal preventive strategies remain uncertain, and evidence comparing closure or reinforcement techniques is limited. Prophylactic mesh use shows benefits in open surgery, but its role in laparoscopy requires further clarification.

**Objective:**

The PRESUME study evaluates whether prophylactic sublay mesh placement at the umbilical trocar site reduces TSIH incidence in patients undergoing laparoscopic cholecystectomy who present at least one established risk factor. Secondary objectives include assessing mesh-related complications and short- and long-term postoperative outcomes.

**Methods:**

This prospective, multicentre, randomised controlled trial provides Level I evidence. Eligible participants are adults scheduled for laparoscopic cholecystectomy with diabetes mellitus, BMI ≥30 kg/m^2^, age ≥65 years, or requiring umbilical incision enlargement. Patients will be randomised 1:1 to standard aponeurotic closure or intraperitoneal sublay mesh reinforcement (Ventralex™ ST). Follow-up visits are scheduled at 30 days, 6 months, 1 year—with ultrasound—and 2 years. The principal investigator will remain blinded during follow-up. The primary endpoint is TSIH incidence; secondary outcomes include surgical site events, postoperative pain, readmissions, and time to return to daily activities. Based on expected TSIH reduction from 35% to 10%, 42 patients per arm are required.

**Discussion:**

TSIH remains a significant postoperative issue with limited preventive evidence in laparoscopy. By assessing the efficacy and safety of prophylactic sublay mesh reinforcement, the PRESUME trial may support future recommendations and improve outcomes in high-risk patients.

## Introduction

An incisional hernia (IH) is defined as a defect in the abdominal wall at the site of a previous surgical incision, detectable through physical examination and/or imaging techniques [1]. In laparoscopic procedures, this entity is further classified as a Trocar Site Incisional Hernia (TSIH) or Port Site Hernia (PSH), depending on the terminology used.

Every year, around 1,000 incisional hernias are operated on in Spain. Despite significant advancements in anesthetic techniques, postoperative care, surgical methods, suture quality, and antibiotic prophylaxis, complications related to abdominal wall closure remain prevalent. These complications include hematomas, surgical site infections, eviscerations, and incisional hernias (IH). They result in hospital readmissions, increased healthcare resource utilization, and higher associated costs. These events negatively affect surgical outcomes and diminish patients’ quality of life.

Numerous studies have advocated for the use of prophylactic mesh during abdominal wall closure in open surgery, demonstrating a reduction in the incidence of postoperative hernias [2]. However, the widespread adoption of laparoscopic surgery since its introduction in 1987 has not eliminated such complications. On the contrary, TSIH/PSH remains the most frequent postoperative complication associated with minimally invasive techniques [3, 4].

It is now recognized that a lack of medium- and long-term follow-up contributes to underdiagnosis of TSIH, particularly since many patients remain asymptomatic or do not seek medical care. Accurate diagnosis often requires a combination of thorough physical examination and imaging modalities such as ultrasound or CT scanning. Recent studies report a surprisingly high incidence of TSIH, ranging from 25.9% in procedures like laparoscopic cholecystectomy to 37.1% in more complex interventions such as laparoscopic colorectal surgery [4–6].

Established risk factors for TSIH include a body mass index (BMI) ≥30 kg/m^2^, age ≥65 years, and enlargement of the umbilical trocar incision [5].

While not all cases of TSIH are diagnosed—largely due to inadequate follow-up—and not all require surgical intervention, the growing use of laparoscopic techniques has rendered trocar site hernias a significant healthcare concern. The most promising strategy to address this issue lies in preventive measures. Although consensus exists among experts on the importance of prevention, relatively few studies have specifically investigated prophylactic strategies. Previous research by our group examined the use of a prophylactic supra-aponeurotic mesh but failed to demonstrate sufficient efficacy in preventing TSIH(7).

### Main and Secondary Hypothesis

The aim of the PRESUME study is to evaluate the effectiveness of a prophylactic sublay mesh in reducing the incidence of TSIH in patients at increased risk. We hypothesize that placement of a prophylactic sublay mesh at the umbilical trocar site in patients undergoing laparoscopic cholecystectomy with one or more established risk factors will significantly reduce the incidence of TSIH compared to standard closure without mesh reinforcement.

Secondary objectives include:

- Quantifying the incidence of complications at the umbilical trocar site
- Assessing the impact of routine prophylactic mesh placement on the incidence of TSIH in high-risk patients
- Evaluating both short-term and long-term complications associated with the use of prophylactic mesh

## Methods and Design

### Ethics and Permissions

This study will be conducted in accordance with the ethical principles outlined in the Declaration of Helsinki and will comply with current data protection regulations, including the European Union’s General Data Protection Regulation (EU GDPR 2016/679). Informed consent will be obtained from all participants prior to enrollment, and all personal data will be securely managed and anonymized.

The institutional review boards of the two participating hospitals have approved this randomized controlled trial. The study protocol has been reviewed and approved by the ethics committee of the coordinating center, Parc Sanitari Hospital Sant Joan de Déu (CEIC approval number: [PS-12-22]) in June 2022, as well as by the institutional review board of the other participating center, Consorci Sanitari del Maresme (CEIC approval number: [31/23]) on May 2023.

### Patient Evaluation and Selection (Inclusion/Exclusion Criteria)

**FIGURE 1:**
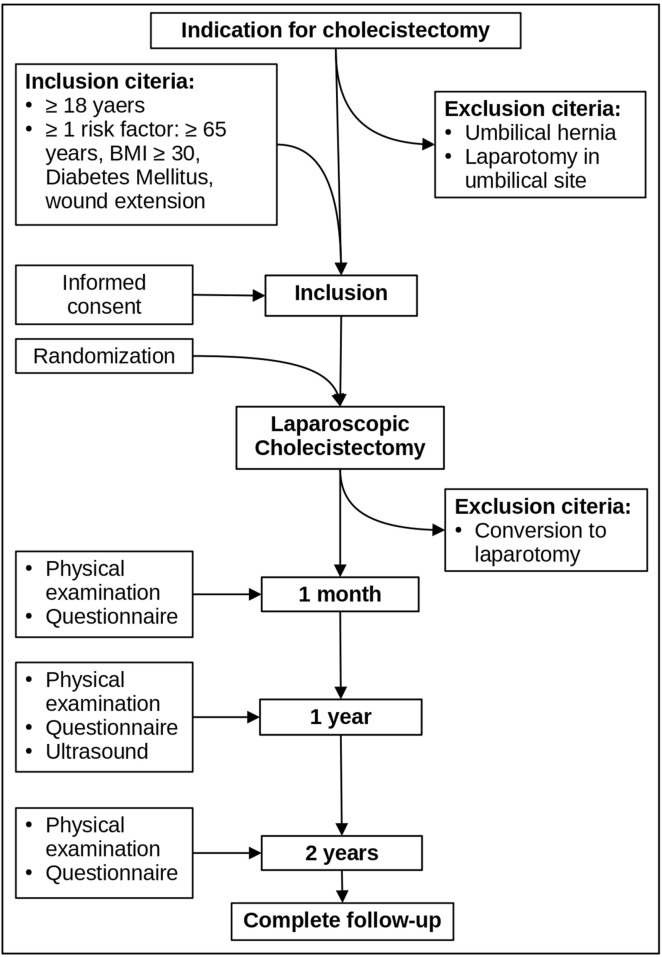
Flow chart.

Patient recruitment will be conducted by the surgeon during routine preoperative consultations. The study will be offered to adult patients (aged ≥18 years) scheduled to undergo laparoscopic cholecystectomy who fulfil at least one of the inclusion criteria. The inclusion criteria comprise the following risk factors for trocar incisional hernia: diabetes mellitus, body mass index (BMI) ≥30 kg/m^2^, age ≥65 years, or intraoperative enlargement of the umbilical trocar incision (Figure 1).

Exclusion criteria include a history of umbilical hernia, prior laparotomy at the umbilical site, or the need for intraoperative conversion to open surgery.

Eligible candidates will receive both verbal and written information regarding the study, including an informed consent document. Participation will be entirely voluntary, with no incentives or coercion involved.

### Randomization Procedure

Participants will be randomized immediately prior to surgery using a computer-generated randomization sequence, in accordance with CONSORT guidelines. Patients will be assigned to one of two groups:

- Control Group: Umbilical trocar site closure will be performed using the standard technique (continuous suture).
- Study Group: A reinforcing intraperitoneal mesh will be placed at the umbilical trocar site following continuous suturing.

### Intervention

The umbilical port will be inserted using the Hasson technique, which requires a transverse incision of aponeurosis. The placement and insertion of additional trocars will be at the discretion of the operating surgeon. The laparoscopic cholecystectomy will be performed in a standard fashion, with the specimen extracted via the umbilical trocar site using an endoscopic retrieval bag. If necessary, the incision may be extended, and the final length of the aponeurotic incision will be recorded.

In the control group, aponeurotic closure will be performed using a continuous suture with 0 polydioxanone. In the study group, a reinforcing mesh will be placed in an intraperitoneal sublay position, and the same continuous 0 polydioxanone suture will be used for mesh fixation. A 4.3 cm composite mesh (Ventralex™ ST Hernia Patch, Bard Davol, New Jersey, USA) will be used in the study group. This mesh consists of an anterior layer of polypropylene monofilament and a posterior absorbable hydrogel barrier, which permits safe contact with the peritoneal cavity. The intraperitoneal sublay position allows for a reduction in surgical time required for mesh placement.

Both groups will receive a single dose of 2 g of cefazolin preoperatively. Local anesthesia will be administered at all trocar insertion sites.

### Follow-Up

Postoperative follow-up will be carried out at 30 days, 6 months, and 1 year after surgery—including abdominal wall ultrasound—and again at 2 years postoperatively.

Each follow-up visit will include assessment of postoperative complications, pain [measured using the Visual Analogue Scale(VAS)], time to return to work, and resumption of daily activities.

### Blinding

The trial is blinded with respect to the principal investigator. Patients are informed about whether a mesh was placed, in accordance with our current protocol. However, the surgeon (principal investigator) remains blinded during follow-up. To maintain blinding, the physical examination must be performed prior to the abdominal wall ultrasound assessment. Subsequently, the ultrasound examination must be conducted by a specialized radiologist.

### Data Collection and Analysis

The primary variable is the occurrence of herniation at the umbilical trocar site (eventration/TSIH).

Secondary variables include:

- Biodemographic data: age, sex
- Comorbidities and clinical history: diabetes mellitus, BMI, hypertension, chronic pulmonary disease, cardiovascular disease, immunosuppression, ASA score, smoking status
- Intraoperative factors: need for enlargement of the umbilical wound, conversion to open surgery
- Postoperative outcomes: surgical site occurrences, pain (measured using the Visual Analogue Scale [VAS]), length of hospital stay, emergency department visits, hospital readmissions, return to work, and resumption of basic activities of daily living

All data will be collected and anonymized through the Research Electronic Data Capture (REDCap ®, Vanderblit University, USA). Access will be restricted to study investigators using individually assigned access credentials.

### Sample Size Calculation

The primary outcome for the sample size calculation is the incidence of umbilical trocar site hernia. Based on published data, the expected incidence of umbilical trocar site hernia in high-risk patients is approximately 35%. The study hypothesises that prophylactic mesh placement will reduce the incidence to 10%; therefore, we assume a 25% reduction.

Assuming a 25% reduction in umbilical trocar site hernia, a two-sided alpha risk of 0.05 and a power of 80% (β < 0.2), and accounting for a 10% dropout or loss to follow-up, the random sample size is 42 patients per group to detect a statistically significant difference between the intervention and control groups.

## Discussion

Incisional hernia is one of the most frequent complications in open surgery, with a considerable rate of morbidity and mortality. Advances in anatomical knowledge and new technological devices have improved surgical techniques and devices aimed at preventing and treating incisional hernias in open surgery.

In recent decades, there has been a significant increase in laparoscopic surgery. Nowadays, laparoscopic access to the abdominal cavity is considered the gold standard for a large number of procedures. Initially, it was assumed that the laparoscopic approach would drastically reduce the incidence and/or severity of postoperative complications related to incisional hernias. However, it is now well recognized that TSIH are highly prevalent. The incidence of TSIH can exceed 30% in patient cohorts with risk factors who are followed up adequately [5]. Such a prevalent pathology—resulting directly from a surgical intervention—warrants special attention through the development of effective preventive measures.

Current guidelines recommend closing all trocar sites that are 10 mm or larger but there is not enough data on optimal closure technique or material [7]. Some researchers have also suggested the use of prophylactic mesh placement as a strategy to prevent TSIH; however, evidence in the literature remains limited [8–10]. There is a scarcity of comprehensive clinical guidelines or meta-analyses that strongly support these practices. Despite the inconclusive nature of the available data, the most recent joint review by the European Hernia Society and the American Hernia Society provided a “weak recommendation” for placing mesh in the preperitoneal space [7]. Previous investigations have yielded conflicting findings: while several studies from the past decade endorsed sublay mesh placement for hernia prevention [8–10], a more recent study assessing onlay mesh did not confirm its efficacy [11]. Based on these findings, and considering that current incisional hernia guidelines recommend sublay mesh placement [12], we hypothesize that positioning a reinforcing sublay mesh (as opposed to a supra-aponeurotic mesh) at the umbilical trocar site in patients with one or more risk factors could reduce the incidence of TSIH.

Finally, an equally important aspect in our current healthcare context is the rationalization of resources. In an effort to highlight the significance of incisional hernias (IH), increase their visibility, and secure funding for research, Poulose et al. published a study estimating the cost of IH repair: $3,873 for outpatient procedures and $15,899 for inpatient surgeries. The study further determined that each 1% reduction in recurrence rates translates into an annual savings of $32 million in procedural costs alone [13].

In our setting, the Catalan Health Service (Servei Català de la Salut, Catalonia, Spain) published in 2013 the official pricing of public health services, listing the cost of a “hernia surgical procedure, excluding inguinal or femoral hernias, in patients over 17 years old, without complications or comorbidities” at a level III reference hospital as €2,608.35 [14].

A cost–benefit analysis focused on reducing the incidence of TSIH represents a valuable avenue for future research.

The objective of our study is to evaluate the efficacy of prophylactic sublay mesh placement in reducing the incidence of TSIH without increasing morbidity. We hypothesize that sublay mesh placement, due to its morphological characteristics and optimal positioning, may be more effective in preventing TSIH.

Our study will provide Level I evidence (Oxford Centre or Evidence- Based Medicine), as it is designed as a prospective, multicenter, randomized controlled trial with 1:1 allocation and double-blinding of patients and principal investigators. Inclusion criteria have been selected to increase the likelihood that participants present the event of interest, as they must have one or more risk factors. Although this may initially appear to limit the generalizability of the results, these risk factors—such as obesity, diabetes mellitus, and advanced age—are highly prevalent in the general population.

## Data Availability

The original contributions presented in the study are included in the article/supplementary material, further inquiries can be directed to the corresponding author.
